# Sleep alters neurovascular and hydrodynamic coupling in the human brain

**DOI:** 10.1073/pnas.2510731123

**Published:** 2026-03-18

**Authors:** Tommi Väyrynen, Johanna Tuunanen, Heta Helakari, Ahmed Elabasy, Vesa Korhonen, Niko Huotari, Johanna Piispala, Mika Kallio, Maiken Nedergaard, Vesa Kiviniemi

**Affiliations:** ^a^Oulu Functional NeuroImaging, Research unit of Health Sciences and Technology, Faculty of Medicine, Medical Research Center, University of Oulu, Oulu 90014, Finland; ^b^Radiology, Diagnostics, Oulu University Hospital, Oulu 90220, Finland; ^c^Clinical Neurophysiology, Diagnostics, Oulu University Hospital, Oulu 90220, Finland; ^d^Center for Translational Neuromedicine, University of Rochester Medical Center, Rochester, NY 14642; ^e^Center for Translational Neuromedicine, Faculty of Health and Medical Sciences, University of Copenhagen, Copenhagen 2200, Denmark; ^f^Biocenter Oulu, Faculty of Biochemistry and Molecular Medicine, University of Oulu, Oulu 90220, Finland

**Keywords:** sleep, fMRI BOLD, cerebrospinal fluid, electrophysiology, fNIRS

## Abstract

Sleep is essential for maintaining brain tissue homeostasis, which is facilitated by enhanced cerebrospinal fluid (CSF) solute transport. Infraslow (<0.1 Hz) vasomotion, CSF flow, and electrophysiological potential all increase during sleep, but their contributions as potential drivers of CSF flow in human brain remain unknown. To investigate this, we measured these signals in healthy volunteers across sleep–wake states using functional MRI blood oxygen level-dependent (BOLD), electroencephalography, and functional near-infrared spectroscopy. We then studied the directed coupling patterns between the three signals, using phase transfer entropy. In the awake state, electrophysiological potential and water concentration changes both predicted hemodynamic BOLD changes across whole brain, reflecting classical functional hyperemia. During sleep, these interactions changed such that the net directionality was lost and the interactions became more bidirectional. Our results show that in addition to neural changes during sleep, nonneural processes such as vasomotor-driven hydrodynamic waves start to gain more impact on human brain activity.

## Significance

Beyond its well-known effects on neuronal activity, human sleep appears also to reorganize the infraslow (<0.1 Hz) oscillation hierarchy in brain. While wakefulness is dominated by unidirectional neurovascular coupling, in which neural activity predicts hemodynamic changes, our study shows that the change in brain state from wakefulness to sleep is accompanied by increased bidirectionality in prediction patterns. Sleep-related increases in signal power were associated with bidirectional prediction patterns involving electrical activity, vascular signals, and water volume changes. These multimodal findings highlight a fundamental change in coupling dependent on brain state, suggesting that both neural and nonneural alteration contribute to sleep-related brain function.

Neuronal activation induces coupled arterial dilatations leading to classical activation hyperemia during the awake state (1). Sleep-associated infraslow oscillations (<0.1 Hz) in the cerebrospinal fluid (CSF) support the clearance of accumulated solutes and metabolites from the cerebrocortical parenchyma (2, 3). Norepinephrine (NE) release from cortical projections of the brainstem locus coeruleus decline in sleep and show concentration oscillations, which induce vasomotor waves in arterial wall myocytes (4, 5). The resultant slow vasomotor waves generate coupled oscillations in cerebral blood volume with inverse CSF volume changes in perivascular spaces (5–7). The NE-driven cortical vasomotion inflates the perivascular CSF space volume and increases the thickness of astrocyte endfeet plastering the outer edge of perivascular spaces of the blood–brain barrier (BBB) (8–10). Numerous human functional MRI (fMRI) studies have demonstrated that vasomotor wave amplitude increases with sleep depth, suggesting a direct link between sleep and vasomotor activity (7, 11–16).

Beyond its impact on vasomotor waves, sleep also enhances infraslow oscillations in the direct current-coupled electroencephalogram (DC-EEG) (17, 18), which further modulate cortical neuronal rhythms over a wide range of frequencies (18–20). Among these rhythms, sigma power has a notable association with the declining phase of locus coeruleus activity during transition to sleep (2, 21) and with memory consolidation after sleep (22). The infraslow EEG oscillations are further connected to changes in cerebral blood volume (CBV), extracellular pH, [K+], and permeability of the BBB (23–25). Recent work has shown that such synchronized neuronal activity facilitates interstitial influx and efflux of electrolytes (26). Synchronous EEG & fMRI studies have also linked infraslow, synchronized neurovascular activity to enhanced solute exchange between interstitial fluid and CSF during sleep (7, 27).

A recent animal study indicated that NE oscillations coordinate vasomotor waves, CSF flow, and EEG sigma power, and that sleep alters their correlation and lag structure (5). Previous studies have often examined these factors in isolation, overlooking their potential mutual interactions. To identify the interactions underlying the sleep-associated increase in CSF flow in humans, we developed noninvasive multimodal imaging protocol not requiring tracers or contrast agents. In particular, we acquired fMRI blood oxygenation level dependent (BOLD) signals sampled at 10 Hz to avoid cardiorespiratory aliasing effects (28). Concomitant electrophysiological changes were measured using high-density DC-EEG, in conjunction with an fNIRS technology with specificity for water to assess CSF volume changes (6, 29). With this multimodal setup, we simultaneously estimated the contributions of vasomotor, electrophysiological, and CSF volume changes in the human brain.

To understand the underlying causal patterns, we studied the interactions between these three signals using information theory-based phase transfer entropy (TE) (30) across awake and sleep states. Prior studies in mice studies have demonstrated hydrodynamic modulation of blood flow oscillations (2, 5, 10). Herein, we investigated whether similar relationships are present in the awake human brain and whether these interactions are altered during sleep.

## Results

**Fig. 1.**
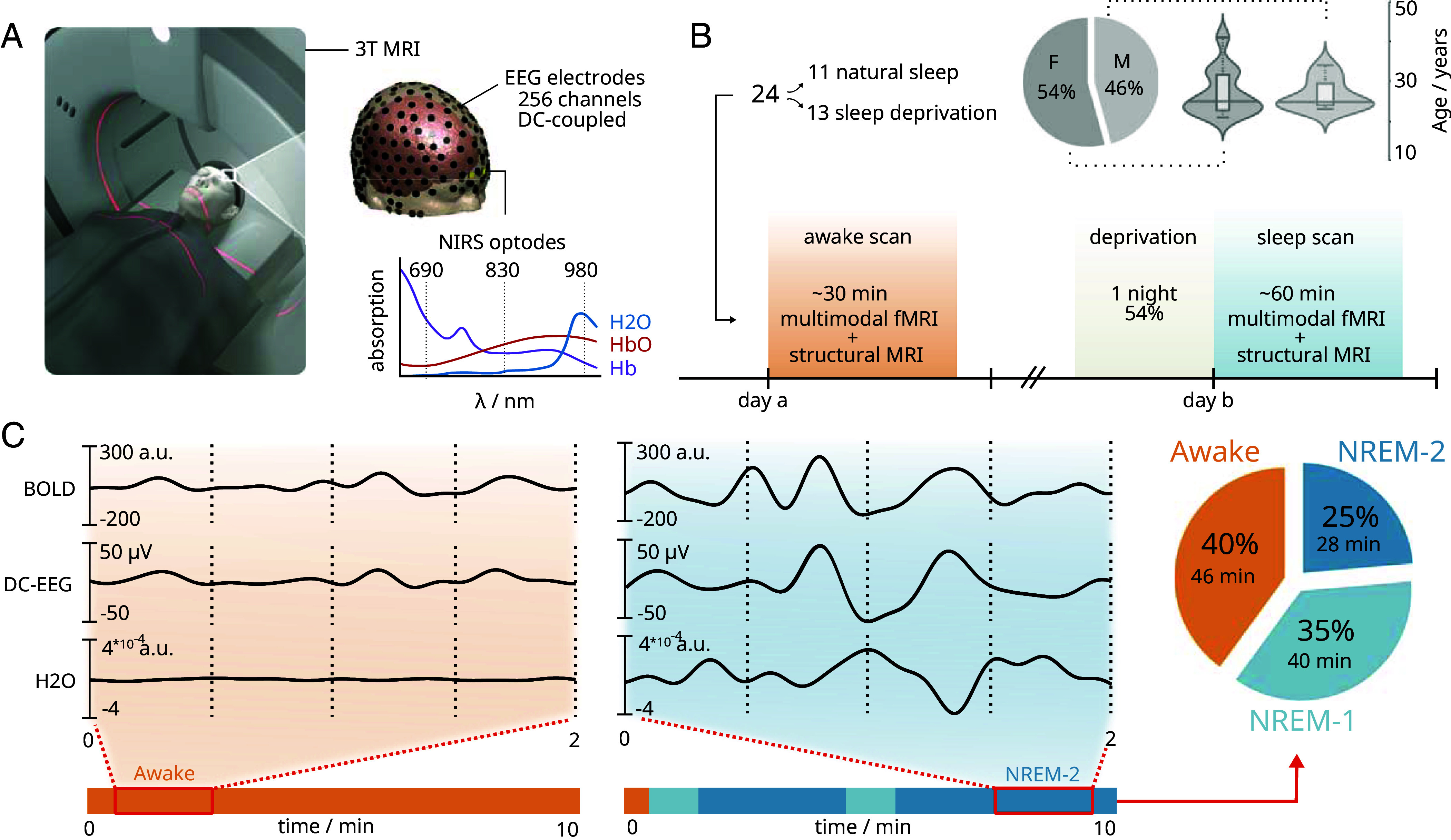
Multimodal imaging protocol for simultaneous measurement of brain water content, electrophysiological, and hemodynamic processes. (*A*) Measurements were conducted in a MRI scanner using a whole-brain magnetic resonance encephalography (MREG) sequence with simultaneous direct-current electroencephalography (DC-EEG) and functional near-infrared spectroscopy (NIRS), which was specifically targeting water concentration changes. (*B*) Participants underwent two scanning sessions comprising functional MREG and structural scans: one during wakefulness and another during sleep. The total scan length consisted of structural scans and 10-min length fMRI scans, which were repeated during sleep scans. The pie chart illustrates the gender composition of the (n = 24) participants, while the violin plot depicts their age distribution. (*C*) Representative signals of infraslow (<0.1 Hz) MREG, EEG, and water NIRS from one participant along with corresponding EEG-derived sleep scoring. For visualization, NIRS signals were calibrated with respect to MREG. The pie chart shows the proportions of gathered epochs of awake, NREM-1 and NREM-2 state across all participants.

To investigate the interconnected hemodynamic, water, and electrical oscillations in the human brain, we employed a multimodal neuroimaging setup (Fig. 1*A*). Our study included 24 healthy participants with an equal gender distribution (54% females) and a mean age of 25 y in both gender groups (Fig. 1*B*). Each participant completed two scanning sessions: one during wakefulness and another specifically targeting sleep. Experienced neurophysiologists (J.P., M.K.) manually classified sleep stages using the EEG recordings. We used these sleep classifications to extract in total 46 min of wakefulness, 40 min of NREM-1 sleep, and 28 min of NREM-2 sleep across all participants during active MRI scanning (Fig. 1*C*).

### Increased Infraslow (<0.1 Hz) Hemodynamic and Electrical Oscillations Occur in Sleep.

**Fig. 2.**
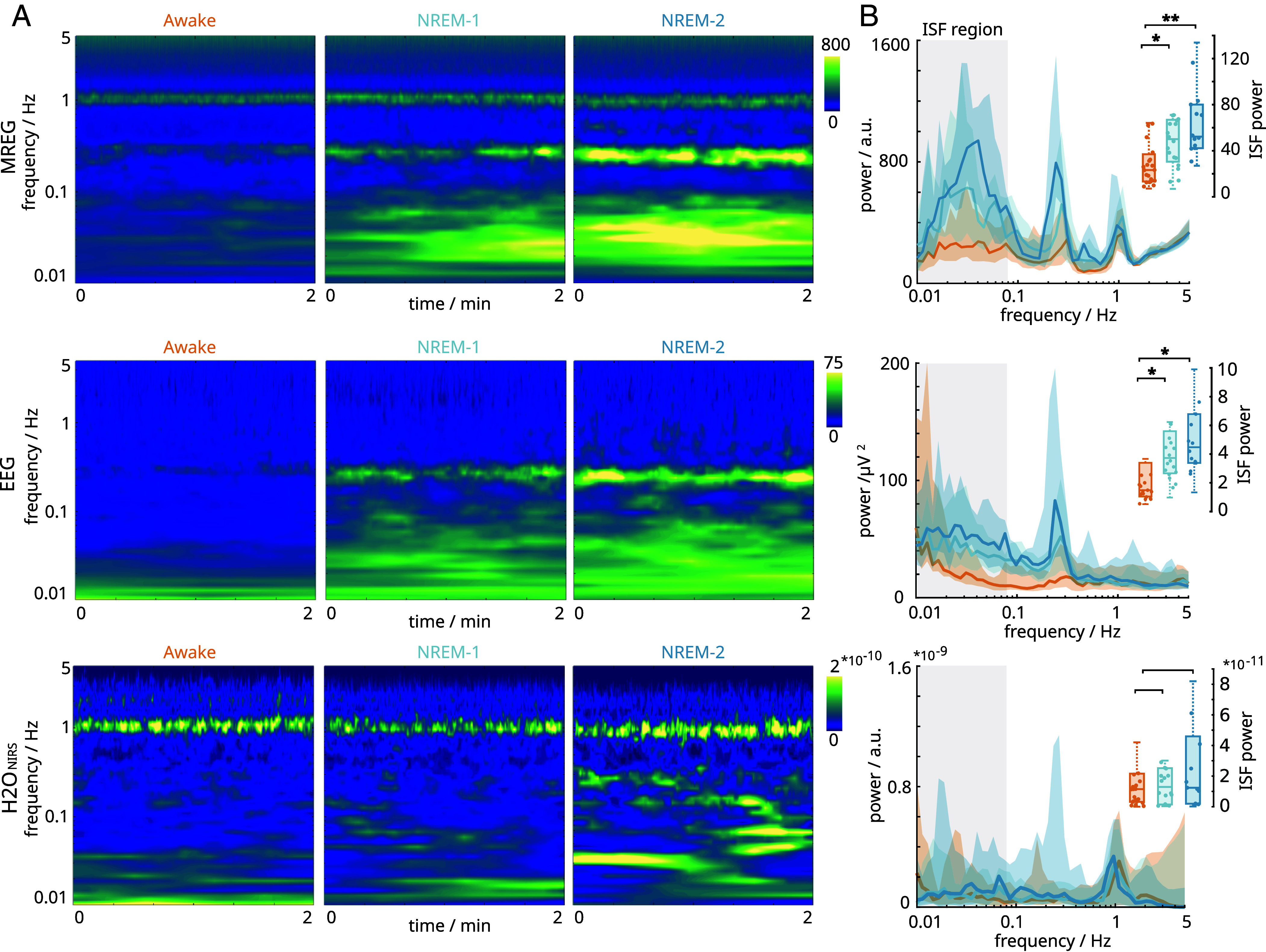
Sleep increases infraslow (<0.1 Hz) hemodynamic and electrical oscillations. (*A*) Time–frequency estimates illustrate the average spectral powers for magnetic resonance encephalography (MREG), electroencephalography (EEG), and functional near-infrared spectroscopy (NIRS) within sleep state-specific epochs. (*B*) Time-collapsed spectral estimates depict power as a function of frequency, where the infraslow frequency range (0.01 to 0.08 Hz) serves for statistical comparison. Data points corresponding to wakefulness are denoted by orange, and nonrapid eye movement sleep by light blue (NREM-1) and dark blue (NREM-2). The solid lines represent the median, while the shaded area indicates the middle 50% of data points. Statistical significance is denoted by asterisks (*p_adj_* < 0.05*, 0.01**, 0.001***). The EEG lacks the cardiac signal due to fMRI-related cardioballistic artifact removal.

We first analyzed the signal behavior in the frequency domain to examine power levels across all modalities during sleep. Using two-min epochs of verified wakefulness and sleep (NREM-1/2), we computed the time-frequency estimates (Fig. 2*A*) within the 0.01 to 5 Hz frequency range. Visual inspection confirmed the presence of three distinct frequency bands: infraslow, respiratory, and cardiac ranges.

In the transition to sleep, there was a clear increase in the infraslow power of MREG signal (Fig. 2*B*), peaking at a frequency of 0.03 Hz. The group mean infraslow power rose from *P_A_* = 23.3 (IQR: 23.6) during wakefulness to *P_N1_* = 50.1 (IQR: 36.3) and *P_N2_* = 52.2 (IQR: 37.8) during sleep (A-N1: *Z_(23,20)_* = −2.59, *p_adj_* < 0.05*; A-N2: *Z_(23,14)_* = −3.46, *p_adj_* < 0.01**). We observed a similar trend in EEG signals, with the infraslow power increasing from *P_A_* = 1.49 (IQR: 2.34) during wakefulness to *P_N1_* = 3.73 (IQR: 2.93) and *P_N2_* = 4.48 (IQR: 3.40) during sleep (A-N1: *Z_(23,20)_* = −2.42, *p_adj_* < 0.05*; A-N2: *Z_(23,14)_* = −2.61, *p_adj_* < 0.05*). However, there were no corresponding changes in the NIRS power measurements across arousal states, with values of *P_A_* = 1.15 (IQR: 1.84)*10^−11^, *P_N1_* = 1.29 (IQR: 2.38)*10^−11^, and *P_N2_* = 1.24 (IQR: 4.40)*10^−11^. Consistent with our expectations and earlier results, power in the infraslow range increased significantly in MREG and EEG measurements in proportion to sleep stage (13, 18).

### The Main Coupling Directions between Hemodynamic, Electrical, and Water Oscillations Change during Sleep.

**Fig. 3.**
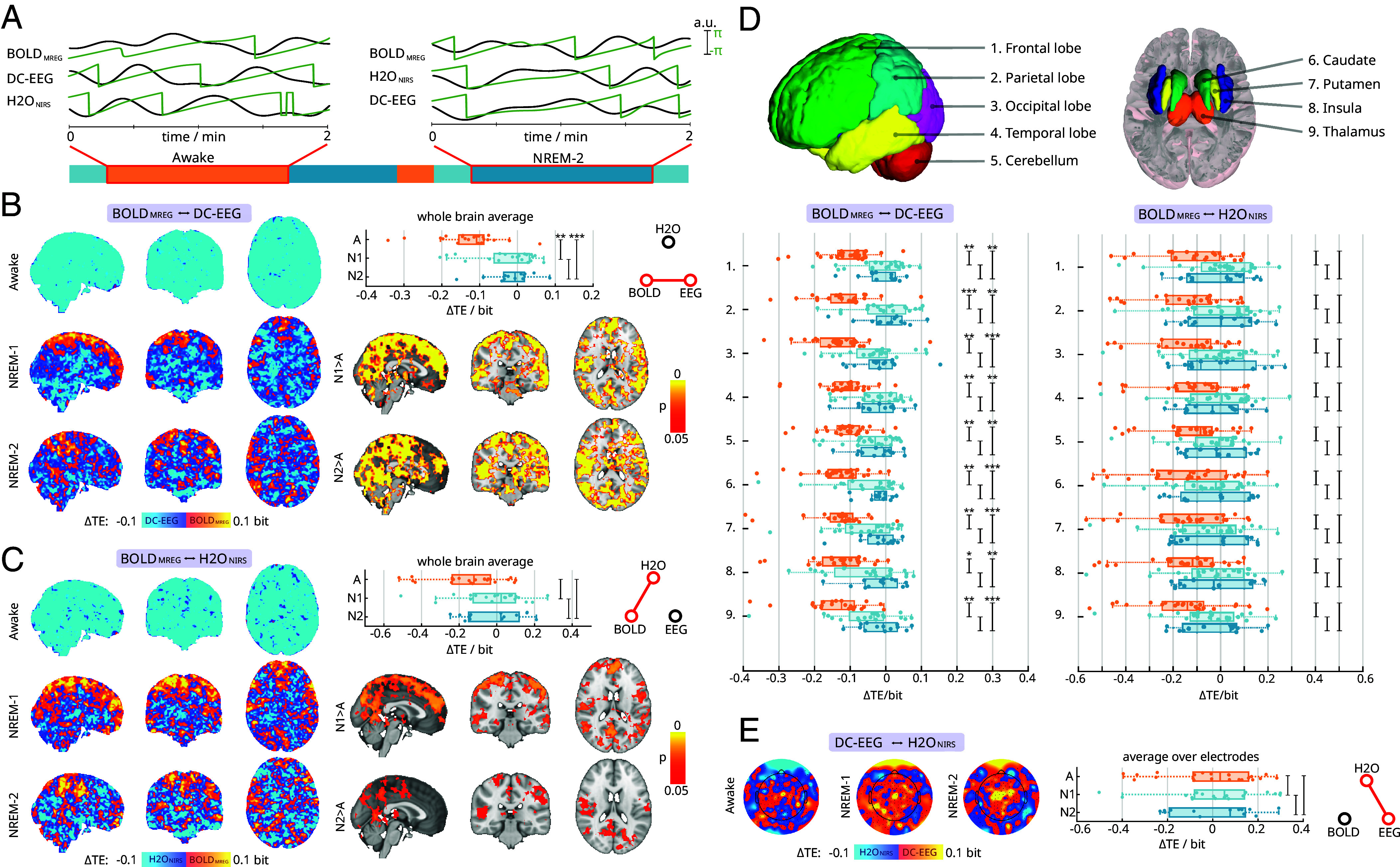
Directed coupling patterns between infraslow brain hemodynamics, electrical, and water fluctuations. (*A*) Transfer entropy in the phase domain (TE) was used to calculate information transfer, utilizing the phase time-series (green) of infraslow signals (black) within sleep state-specific epochs. The sign of *ΔTE* indicates the main direction of information transfer, as also highlighted in the color bars. (*B*) Blood oxygenation level-dependent (BOLD_MREG_) and electroencephalography (EEG) coupling patterns were assessed in wakefulness and nonrapid eye movement (NREM)-1/2 sleep. Positive values correspond to BOLD_MREG_ prediction of EEG, and negative values vice versa. We calculated the average TE over the whole-brain volume, where asterisks denote significance of differences (*p_adj_* < 0.05*, 0.01**, 0.001***). Below, the spatial distributions of statistically significant areas (*p_adj_* < 0.05) are shown in comparison to awake state. (*C*) Similarly, we present TE between BOLD_MREG_ and cortical water concentration changes (H2O_NIRS_). Here, positive values indicate BOLD_MREG_ prediction of H2O_NIRS_, and negative values vice versa. We also present the whole-brain averages for BOLD_MREG_↔H2O_NIRS_ coupling and spatial distribution of voxel wise differences. (*D*) Regional TE analysis within regions of interest (ROI) for BOLD_MREG_, EEG, and H2O_NIRS_. The box plot depicts the average TE within the ROI. (*E*) Topographies show average TE patterns between EEG and H2O_NIRS_ taken over the subjects, whereas the box plot depicts average TE over the electrode space.

Based on recent observations in mice (5), we hypothesized that infraslow BOLD_MREG_ signal would be linked to electrophysiological DC-EEG and macroscopic water fluctuations H2O_NIRS_. To study the coupling patterns, we calculated the information transfer among these signals using phase transfer entropy (TE) (Fig. 3*A*), where TE = 0 bits indicates no connection between the phase time series.

We found that electrophysiological EEG changes measured during wakefulness predicted the infraslow fluctuations in BOLD_MREG_ signal (Fig. 3*B* and *SI Appendix*, Fig. S1) as expected from basic principles of neurovascular coupling. Furthermore, the water fluctuations in H2O_NIRS_ signals also predicted the BOLD signal (Fig. 3*C* and *SI Appendix*, Fig. S1, shown in blue colors) across whole brain, as indicated by a coherent negative net information transfer. In NREM1-2 sleep states, the BOLD_MREG_↔EEG and BOLD_MREG_↔H_2_O_NIRS_ coupling patterns both were altered significantly with respect to awake state (Fig. 3 *B* and *C*). The voxel level analysis showed opposing average coupling directions on cortical brain with respect to deeper subcortical brain regions.

Spatially, the area with significantly (*p_adj_* < 0.05) altered BOLD_MREG_↔EEG prediction spanned a wider cortical area than the smaller but overlapping BOLD_MREG_↔H2O_NIRS_ prediction (Fig. 3 *B* and *C*). Within these significantly altered prediction regions, 81% of voxels showing increased BOLD_MREG_↔H2O_NIRS_ prediction in NREM-1 also showed a significant increase of BOLD_MREG_↔EEG, whereas there was 98% overlap for NREM-2 sleep. We further investigated if interregional delays in BOLD_MREG_ signal could explain the observed differences in cortical and subcortical prediction values. We tested this by studying voxel-wise delays within BOLD_MREG_ signal with respect to the frontal seed (*SI Appendix*, Fig. S2*A*). The mean delay maps did not correlate significantly with TE values (*SI Appendix*, Fig. S2 *B* and *C*). These results suggest that the observed coupling patterns did not arise from differences in these static correlation structures.

The whole brain *ΔTE* averages during the awake state supported the voxel level results, being clearly net negative. During sleep these averages shifted to around zero median, indicating no dominant prediction in either interaction direction. The whole brain average TE values for BOLD_MREG_↔EEG (Fig. 3*B*) shifted from *ΔTE*_A_ = −0.11 bit (IQR: 0.07 bit) during wakefulness to *ΔTE*_N1_ = 1.8*10^−3^ bit (IQR = 0.09 bit) during NREM-1 and *ΔTE*_N2_ = −0.01 bit (IQR = 0.05 bit) during NREM-2. These shifts reflected statistically significant increases (A-N1: *Z*_23,20_ = −3.4, *p_adj_* < 0.01**; A-N2: *Z*_23,13_ = −3.7, *p_adj_* < 0.001***; N1-N2: *Z*_20,13_ = −0.06, *p_adj_* = 0.96). Similarly, BOLD_MREG_↔H_2_O_NIRS_ coupling demonstrated an increasing trend over the whole-brain averages (Fig. 3*C*), without reaching significance. The median information transfer was *ΔTE*_A_ = −0.12 bit (IQR = 0.20 bit) during wakefulness, which increased to *ΔTE*_N1_ = 0.02 bit (IQR = 0.22 bit) during NREM-1 and *ΔTE*_N2_ = 0.02 bit (IQR = 0.27 bit) during NREM-2 sleep. These cortically dominant changes were not significant for the whole brain averages: (A-N1: *Z*_22,20_ = −2.4, *p_adj_* = 0.06; A-N2: *Z*_22,13_ = −1.6, *p_adj_* = 0.18; N1-N2: *Z*_20,13_ = 0.4, *p_adj_* = 0.87).

We hypothesized that cortical and subcortical brain regions having opposing net interaction directions in voxel level averages would cancel each other out, thus resulting in zero whole brain average TE. We further investigated the regional changes, utilizing region of interest (ROI) analysis. With BOLD_MREG_↔EEG (Fig. 3 *D*, *Left*), the net information transfer within all nine ROIs increased significantly between awake and sleep states. This effect was evident between awake-NREM1 and awake-NREM-2 states (*SI Appendix*, Table S1). Similarly, with BOLD_MREG_↔H2O_NIRS_ (Fig. 3 *D*, *Right*) awake state net prediction was negative within all ROIs, pointing to H2O_NIRS_ prediction of BOLD_MREG_ signal, whereas during sleep, the median centered around zero. In BOLD_MREG_↔H2O_NIRS_, there were no significant changes within the ROIs with respect to arousal state. Contrary to our expectations, the ROI analysis did not show reversed interactions directions in any of the ROIs studied during sleep, but rather indicated uniform coupling dynamics across the brain.

To further investigate the cause of the sleep-related shift in net coupling directions, we extracted the information transfer values in both interaction directions i.e., TE(x→y) and TE(y→x). By studying the separated TE values, we could then ascertain if the loss of net drive can be explained by increased information transfer in one direction, or conversely by decreased information transfer in the other direction. Our results revealed that overall information transfer in all three coupling patterns remained quite stable with respect to arousal state (*SI Appendix*, Fig. S3). The loss of BOLD_MREG_↔EEG net prediction direction during sleep seemed to arise from the combined effect of reduced information transfer of EEG→BOLD_MREG_ and concurrent increase in BOLD_MREG_→EEG information transfer. Similarly, loss of net prediction direction in BOLD_MREG_↔H2O_NIRS_ coupling resulted from both factors, i.e., reduced H2O_NIRS_→BOLD_MREG_ and increased BOLD_MREG_→H2O_NIRS_ information transfer.

We then focused on the final interaction pair: EEG↔H2O_NIRS_. Here, the topographic analysis demonstrated more stable coupling patterns with respect to arousal state as compared to the other interactions pairs (Fig. 3*E*). Generally, the central upper vertex electrodes showed continuous prediction of EEG over H2O_NIRS_, while more peripheral, distal electrodes presented an opposite prediction: H2O_NIRS_→EEG. The average *ΔTE* over all electrodes showed that the prediction EEG→H2O_NIRS_ was predominant, without significant alterations with respect to arousal state (Fig. 3*E*): (A-N1: *Z*_22,20_ = −0.14, *p_adj_* = 0.9; A-N2: *Z*_22,13_ = −0.29, *p_adj_* = 0.9; N1-N2: *Z*_20,13_ = 0.13, *p_adj_* = 0.9).

Altogether, we found that infraslow brain hemodynamics, water volume changes, and electrophysiological changes are directionally coupled. Our results showed that the awake state is the most coherent, where water dynamics and electrophysiological brain changes both predict hemodynamic signal changes throughout entire brain. In NREM-1/2 sleep, the net prediction direction was lost due to simultaneously increased hemodynamic drive and decreased water and electrophysiological contribution.

### The Sleep-Associated Increased Speed and Power of the Infraslow BOLD Overlaps with Altered Coupling Patterns in Primary Sensory Cortices.

**Fig. 4.**
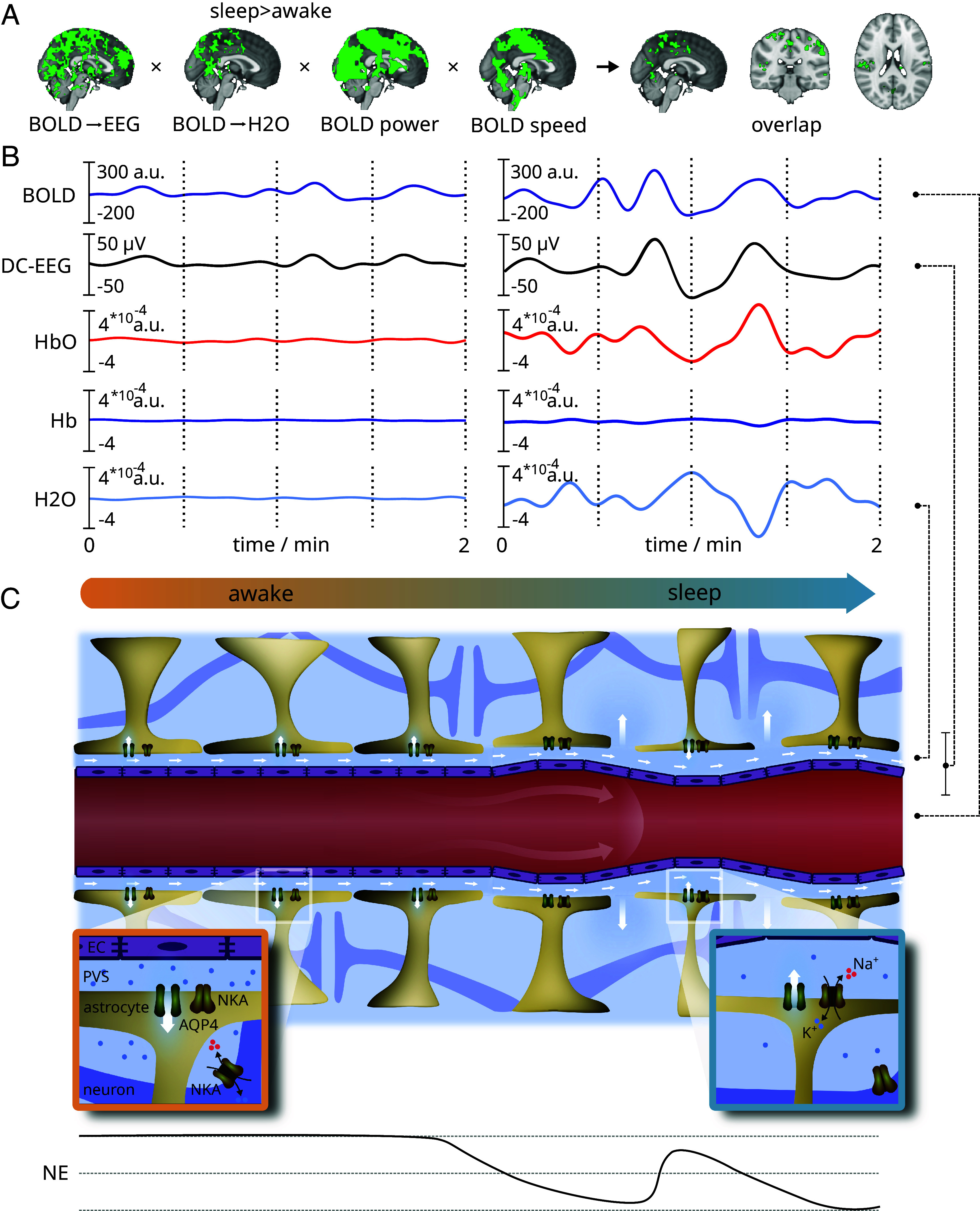
Spatially overlapping changes in coupling, power, and speed of blood oxygen level-dependent (BOLD) oscillations, and the hypothesized cellular mechanisms. (*A*) Multimodal evidence of spatial overlap in primary sensory and motor areas, showing significant increases in sleep induced infraslow (<0.1 Hz) BOLD pulsation power, wave propagation speeds (31), and changes in coupling of BOLD with both water dynamics and electrophysiological brain activity. (*B*) Concurrent infraslow signals, capturing BOLD signal, electroencephalography (DC-EEG), and functional near-infrared spectroscopy (fNIRS) during wakefulness and sleep. BOLD-calibrated, synchronous fNIRS signals indicate that the water (H_2_O) signal is anticorrelated with arterial HbO_2_ and BOLD signal (5, 6, 29). Changes in venous Hb concentration are less pronounced, consistent with animal studies suggesting that BOLD signal generation is driven more by speed than by volume changes in the venous compartment (32). (*C*) A theoretical illustration of regional electro-, hydro-, and hemodynamic interactions in wakefulness and NREM sleep. During NREM sleep, reduced orexin levels induce slow oscillations in norepinephrine (NE) levels (5, 33) and extracellular potassium [K+] concentration (34). These oscillations drive vasomotor waves detected by both BOLD and oxygenated hemoglobin (HbO_2_) signals (13, 14, 32), which are accompanied by anti-correlated H_2_O waves, as in recent animal findings (5, 10). Low NE levels reduce astrocytic volume by decreasing Na^+^/K^+^-ATPase (NAK) activity (3, 5). Pulsatile vasomotor waves cause oscillations in astrocytic endfeet and perivascular volumes (10). These oscillations facilitate hydrodynamic interstitial/cerebrospinal fluid (I/CSF) exchange via enlarged interastrocytic gaps formed by astrocytic shrinkage. This mechanism provides in theory a generation mechanism for the slow but high-voltage infraslow oscillations in EEG. Finally, the NE oscillations drive an important, increasing effect on neuronal NAK-channel activity that is opposed to the astrocytes effect closer to perivascular space. This opposing effect can explain the previously detected electro-osmotic potential difference increasing the I/CSF exchange over glia limitans explaining partially also the high-voltage DC-EEG potential (26).

We have previously shown with BOLD_MREG_ scans that pulsation power and speed both increase during NREM-sleep (13, 31). Here, we further explored whether these changes BOLD_MREG_ characteristics are associated with the same brain regions where the sleep-related changes in BOLD_MREG_ prediction patterns had occurred. Indeed, the increased power and speed of BOLD_MREG_ changes did overlap with voxelwise maps of the same brain regions where we observed altered coupling patterns, especially in the primary sensory cortical brain regions, posterior insula, thalamus, and upper cerebellum (Fig. 4*A* and *SI Appendix*, Fig. S4). The spatial overlap suggests either that vascular flow is less restricted in these areas or that the increased pulsation power imposes a faster flow through the porous brain tissue.

## Discussion

In this study, we used a noninvasive multimodal neuroimaging setup to investigate the brainwide coupling of infraslow (<0.1 Hz) brain oscillations of the human brain including vasomotion (BOLD_MREG_), electrophysiological signals (DC-EEG), and CSF fluctuations (fNIRS) of healthy volunteers. During the awake state, low-power infraslow electrophysiological potential and CSF oscillations both predicted vasomotor BOLD waves, consistent with neurovascular coupling (32, 35). In contrast, during sleep, the infraslow power levels increased, and the main coupling directions seen during awake state were lost. Instead, the interactions became more bidirectional, canceling out the net drive. Considering recent literature reports (2, 5), we conclude that our findings link to increased modulatory effect of vasomotor waves over CSF flow and electrophysiological oscillations in human NREM-sleep.

### Hemodynamic Changes Are Coordinated by Electrophysiological and CSF Changes in Awake Human Brain.

In the awake condition, neural activation is coupled to slower increases in regional blood flow, which are traditionally thought to supply more oxygen and glucose in response to increased metabolic demand (1). However, recent findings suggest that synchronized neuronal activation following blood flow hyperemia also facilitates glymphatic solute transport and clearance, where the vasodilatory pulsations could potentially serve as an additive mechanism to accommodate increased metabolic demands (7, 26, 36–38). Consistent with this proposition, coupling patterns in awake brain suggested sequential drive pattern in which electrophysiological activity and cortical water movement predicted BOLD changes: DC-EEG & H2O → BLOOD (Fig. 3), as has been also demonstrated in mice (5).

The water movement follows upon the neuronal [K+] release that is buffered by astrocytic K^+^_IR4/5_ channels, with further facilitation of passive fluid flow by neighboring astrocytic AQP4 water channels (39). The local [K+] increases are also sensed by capillary endothelia K^+^_IR4/5_-channels, which may facilitate upstream vasomotor dilatations via inward hyperpolarizing waves mediated by gap junctions along blood vessels (40). The occurrence of inward waves triggered by local [K+] increases offers a direct mechanism whereby NE effects on ion channels drive vascular vasomotor waves.

### Reduced Coordination of Brain Hemodynamics during Human NREM-Sleep.

In sleep, the vasomotor waves were no longer driven by electrophysiological changes or by cortical water volume fluctuations. The loss of dominant direction in the coupling was associated with increased hemodynamic prediction over the electrophysiological and cortical CSF volume changes and a concurrent decrease of CSF and electrophysiological prediction over hemodynamics (*SI Appendix*, Fig. S3). As the information transfer baselines remained relatively constant during sleep, this implies that the interactions became more bidirectional, rather than simply decreasing the amount of overall drive. Thus, it is plausible that the direction of underlying causality oscillates over time, where the drivers take turns in providing the predominant modulation.

One potential trigger for the directionality change with time could be oscillating NE-levels. In sleep, the low level of NE release enables an increased relative activity of the Na^+^/K^+^-ATPase in astrocytes, while reducing its activity in neurons (34, 41). These opposing responses of astrocytic and neuronal Na^+^/K^+^-ATPase activity could theoretically generate an intercellular electrophysiological potential, resulting in osmotic pressure differences that would drive CSF water flow (5, 26, 34). As NE-levels slowly oscillate at ~0.02 Hz, the resulting opposite NE effects in the astrocytes and neurons could explain the increase in bidirectional effects observed during sleep (Fig. 4).

Along with oscillating NE-levels, the thickness of astrocytic endfeet at the BBB and perivascular space volume also oscillate, which could together facilitate the hydrodynamic electrolyte oscillations over the BBB *glia limitans* to extend further into perivascular spaces via widened interastrocytic gaps (10, 18, 20). These NE-driven vasomotor oscillatory mechanisms could underlie the increased power of DC-EEG oscillations during reduced neuronal activity in sleep found herein and as reported previously (17, 18) and could account for the increased synchrony between DC-EEG and neuronal activity during sleep (18–20) in addition to the increased bidirectionality.

These findings, combined with recent literature reports, suggest that global vasomotor/hydrodynamic interstitial fluid oscillations under the control of NE increase during sleep, while the coupling between regional neuronal activity and hemodynamic fluctuations changes. A key factor underlying this could be marked oscillatory changes due to the bidirectional effect of global NE on neuronal and astrocyte Na^+^/K^+^-ATPase activities.

### Pulsation Power and Speed Increases Overlap with Decreased Net Drive during Sleep.

Consistent with previous findings (11–13, 15, 42), our human MREG results also showed an increase in infraslow BOLD oscillation power. Previous work has likewise shown that vasomotor waves drive the movement of injected CSF tracers within arterial wall structures (43). Furthermore, the intracranial propagation speed (31) of the vasomotor BOLD waves increases in sleep, especially in the primary sensory and motor regions, indicating an acceleration of brain water movement (Fig. 4 and *SI Appendix*, Fig. S4). Notably, neuronal slow delta activity also increases in the same regions, which previous animal experimentation has linked to enhanced CSF solute transport via widened interstitial spaces (13, 44). Furthermore, the electrophysiological drive of brain water tends to increase in the same parietal areas during sleep (Fig. 3*E*). Our results suggest that brain water increasingly flows during sleep under the drive of infraslow electrical oscillations (26).

Invasive animal experiments show that vasomotor waves are driven by infraslow fluctuations in extracellular NE levels released from projections of the locus coeruleus (2, 21), causing anticorrelated blood and CSF oscillations (5, 6). The descending phase of NE activity also correlates with the occurrence of sleep spindles and enhanced glymphatic clearance of tracers (2, 5, 21). Infraslow vasomotor waves detected in the BOLD_MREG_ (45–48) signal also reflect reciprocal oscillations in tissue blood signal and CSF (5, 29, 45). Similarly, Hb/HbO2 levels. i.e., cerebral blood volume changes of fNIRS signal, anticorrelate with brain H_2_O volume changes (6). These multimodal lines of evidence concur in indicating that the opposing signals all arise from different compartments.

Large vasodilatory oscillations in intracranial blood volume, simultaneously captured here from BOLD signals and the arterial HbO2 signal in fNIRS (Fig. 4*B*), were associated with opposing water concentration changes, consistent with the Monro-Kellie doctrine, stating the sum of brain tissue, CSF, and intracerebral blood volumes must stay constant (5–7, 10, 29).

### Limitations and Future Directions.

Contrary to our expectations, the infraslow CSF oscillations measured by our H2O_NIRS_ setup did not increase during sleep. We suppose that this may be due to several methodological factors: Infrared light passes through several water compartments, which could contribute to the net water fNIRS signal. Anatomical variations across participants such as skull thickness, air sinus spaces, and CSF spaces under the frontal skull bone also directly affect the photon path length, thus altering signal sensitivity. The technical lack of multiple signal sources in fNIRS prohibit the use of averaging strategies, which might have greatly improved the signal-to-noise ratio. Due to optode placement of the fNIRS optodes, the water dynamics observed in this study are most accurately depicted in frontal brain regions, although we expect the CSF dynamics in the subarachnoid space to correlate on a larger scale.

Connectivity estimates can return spurious estimates in the presence of common drivers. In line with this, results of our previous investigation of the relationship between infraslow EEG and faster neural processing did not favor neural cortical changes as a common driver of infraslow EEG. The investigation of other possible common drivers remains a matter for future studies, where multivariate TE with increased spatial coverage of water dynamics could provide a useful framework. Future studies are needed to more thoroughly assess the effect of BOLD lag structure on signal prediction values. Furthermore, the use of longer data epochs may allow for the detection of more nuanced coupling effects and enable the examination of temporal changes in signal prediction patterns.

## Materials and Methods

Experimental setup. This study was approved by Regional Ethics Committee of the Northern Ostrobothnia Hospital District. We obtained written informed consent from all participants in accordance with the Declaration of Helsinki. The datasets utilized in this study were originally gathered as part of our prior investigation (13, 14, 18) involving 24 healthy controls (13 females, 11 males). Participants underwent scanning twice across separate sessions: once during wakefulness and once during sleep. The total duration of awake scans was approximately 30 min, while the sleep scans lasted for about an hour. The measurement protocol included structural sequences and fMRI MREG sequences lasting 10 min each, which were repeated during sleep scans. To increase sleep pressure and enabling faster onset of the sleep recordings, 13 subjects underwent one night of sleep deprivation, which was monitored with smart rings (Oura Health Oy). For each participant, 2-min data epochs were extracted, as these represented the longest continuous segments available across subjects and sleep stages. Data outside these epochs were excluded to avoid unequal weighting across participants. Our study employed a multimodal neuroimaging setup (49) including an MRI scanner to capture functional ultrafast MREG and structural image series. Spontaneous brain activity was recorded with EEG, which was further used to derive sleep state–specific information. Sleep scoring was performed in 30-s epochs by experienced neurophysiologists (J.P., M.K.) according to AASM criteria. Functional NIRS was used to measure changes in macroscopic water concentrations. All modalities were synchronized with the MRI scanner’s optical timing pulse. Calculations were performed using MATLAB (v.R2023b, MathWorks).

### MRI Acquisition and Preprocessing.

Functional and structural images were acquired using a Siemens MAGNETOM Skyra 3T scanner equipped with a 32-channel head coil. For structural 3D MPRAGE scanning (TR = 1,900 ms, TE = 2.49 ms, TI = 900 ms, FA = 9°, FOV = 240 mm), we used a slice thickness of 0.9 mm. Functional imaging was performed using an ultrafast MREG-sequence (TR = 100 ms, TE = 36 ms, FA = 5°, FOV = 192 mm), which employs k-space undersampling to reach a sampling frequency of 10 Hz with voxel size of 3 mm (50). We set a crusher gradient to 0.1, which was optimized for detecting physiological signal sources, while mitigating slow drifts and stimulated echoes.

Reconstruction of MREG images involved the utilization of L2-Tikhonov regularization, wherein a lambda value of 0.1 was determined using the L-curve method (51). Dynamic off-resonance correction in k-space was implemented to reduce B0-field artifacts and mitigate respiratory motion. Image preprocessing followed the standardized FSL (Functional MRI of the Brain’s software library) preprocessing pipeline (52). A high-pass filter was used with a cut-off frequency of 0.008 Hz. Subsequently, we performed motion correction followed by brain extraction. To eliminate artifactual spikes, datasets were despiked (53). The structural 3D MPRAGE images were used in the registration of functional datasets into the MNI152 standard space (Montreal Neurological Institute). Continuous 2-min segments of wakefulness and NREM-1/2 sleep were identified using EEG-derived sleep scores (14).

### EEG Acquisition and Preprocessing.

EEG recordings were acquired using a GES 400 (Magstim EGI) system, which included a direct current-coupled amplifier (Net Amps 400), and a high-density 256-electrode system (HydroCel Geodesic Sensor MR net) with the electrode “Cz” serving as the reference channel. The recordings were conducted at a sampling rate of 1 kHz, except for three sleep and five awake subjects in whom the experimentalists had inadvertently selected 250 Hz. Prior to measurements, we performed a visual inspection of signal quality and electrode impedances.

The initial steps in data processing involved the removal of gradient and ballistocardiographic artifacts through template subtraction (54, 55); Brain Vision Analyzer v.2.1, Brain Products). Subsequently, bad channels were excluded based on the following criteria: SD exceeding 2,000 µV, average correlation with neighboring electrodes falling below 0.1, or electrode impedance surpassing 1 MΩ. Upon identifying bad channels, spherical interpolation was used to replace the removed channels (56). EEG signals were decimated to 10 Hz to align with the sampling frequency of other modalities.

### NIRS Acquisition and Preprocessing.

Our functional NIRS device (29) utilized a frequency-coding technique in which the emitted light was modulated at specific frequencies for each wavelength. High-power LEDs generated monochromatic light at wavelengths of 690, 830, and 980 nm, facilitating measurements of Hb, HbO, and H_2_O, respectively. Specifically, we selected the 980 nm wavelength for its high absorbance of water, ensuring high sensitivity in water dynamics measurement. At the receiver optode, the light was demodulated again with the corresponding frequencies. To maintain consistent optode positioning, we separated the source and receiver optodes on both sides of the EEG electrode “Fp1” at a fixed distance of 3 cm, thereby ensuring a constant distance between measurements, while enabling near-infrared light to reach the cerebral cortex (29).

Initially, NIRS transmittance values were used to compute HbO, HbR, and water concentrations following the modified Beer–Lambert law (29, 57). NIRS concentration signals were decimated to 10 Hz to match the sampling rates of all three modalities.

### Estimation of Spectral Properties.

We employed complex Morlet wavelets in wavelet convolution to conduct time–frequency spectral analysis (Fig. 2 *A* and *B*). These wavelets consist of sine waves at various frequencies tapered by a Gaussian window, thereby providing temporal specificity. Convolving time series with each frequency’s wavelet transforms the data into frequency-specific power by computing the squared magnitude of the convolution results. Unlike rectangular windowing, the Gaussian window reduces ripple effects typically associated with sharp edges in the kernel signal. We chose wavelet convolution due to its ability to focus on time-domain changes, its computational efficiency, and its adherence to the assumption of stationarity, contrary to conventional spectral methods (58). We applied a logarithmic frequency range extending from 0.01 Hz to 5 Hz with 50 steps, having selected the 5 Hz upper limit as it represents the Nyquist frequency of the MREG recordings. To mitigate edge effects that could potentially contaminate the time series, particularly in low-frequency filtering, we implemented the mirroring technique. The number of wavelet cycles (N = 8) was held constant, regulating the trade-off between frequency and temporal precision. Given that we had nearly 70,000 spatially correlating signals in each MREG dataset, we employed a simple random sampling technique without replacement to reduce computational cost. Here, we used 5% (3,405 voxels) of all voxels, ensuring that each brain voxel had an equal probability of being chosen. Finally, we calculated power in the frequency range of 0.01 to 0.08 Hz using integral approximation and rectangular windowing. We averaged the spectral power estimates over the MREG voxels and EEG electrodes to obtain global spectra.

### Inferring Directed Coupling Patterns.

Directed coupling patterns were analyzed in the infraslow frequency range, using the 2-min sleep state–specific epochs (Fig. 3). With sampling rates matched across modalities, we constructed a finite impulse response (FIR) filter with Hamming windowing, filter order 3,000, and cutoff frequencies 0.01 and 0.08 Hz to bandpass filter the signal into the infraslow frequencies. The chosen frequency band allowed the inclusion of slow hemodynamic processes, but leaving out potential slow respiration effects and faster cardiac effects. The mirroring method was used to ensure that no edge effects remain after temporal filtering, and zero-phase recursive filtering was used to avoid phase offset and distortions.

Standard correlative metrics can fail to inferaccurately the directed interactions, especially in the presence of nonlinear interactions. To address this limitation, we used phase transfer entropy (TE), a nonlinear extension of the classical Granger causality, to quantify putative causal interactions among physiological signals (30). TE is a dynamic and directed measure of information transfer (30, 59). Essentially, TE represents the average information from a source signal that aids in predicting the next value of the target concerning its past. We calculated TE in the phase domain, which has been shown to be more robust in comparison to real valued TE in the presence of environmental noise (30). We applied Hilbert transform for the infraslow signals to compute the analytical signals $z_{\mathrm{ISF}}\left(n\right)$. From these analytical signals, we extracted the instantaneous phases as $\theta_{ISF}=\;arg\left(z_{\mathrm{ISF}}\left(n\right)\right)$.

In calculating TE, we chose a discrete estimator to represent the state space, being the simplest form of estimators. Marginal entropies, joint entropies, and phase transfer entropies were defined as

$$H\left(\theta_{y}\left(t^{\prime }\right)\right)=-\sum p\left(\theta_{y}\left(t^{\prime }\right)\right)\log p\left(\theta_{y}\left(t^{\prime }\right)\right),$$

$$H\left(\theta_{y}\left(t^{\prime }\right),\theta_{x}\left(t^{\prime }\right)\right)=-\sum p\left(\theta_{y}\left(t^{\prime }\right),\theta_{x}\left(t^{\prime }\right)\right)\log p\left(\theta_{y}\left(t^{\prime }\right),\theta_{x}\left(t^{\prime }\right)\right),$$

$$\begin{array}{c} {\mathrm{TE}}_{x\to y}=H\left(\theta_{y}\left(t\right),\theta_{y}\left(t^{\prime }\right)\right)+H\left(\theta_{y}\left(t\prime \right),\theta_{x}\left(t^{\prime }\right)\right)-H\left(\theta_{y}\left(t^{\prime }\right)\right)\\ \;-H\left(\theta_{y}\left(t\right),\theta_{y}\left(t\prime \right),\theta_{x}\left(t^{\prime }\right)\right), \end{array}$$

where *t’* refers to past time points *t’* = t-δ, and δ is the analysis lag.

If the source–target delay is set to maximize information transfer, it aligns with the actual causal delay under simple conditions. However, predictive information can be accurately estimated over a wide range of lags (30). Here, we assumed a constant delay of one cycle, in consideration of the computational cost of identifying the maximal information transfer. We calculated pairwise TE for each MREG voxel, including all EEG electrodes and water concentration signals. TE values were then averaged over the EEG electrode space and for whole brain averages also over the voxel space, aiming to reduce data dimensionality. We used FSLs MNI structural atlas to divide the brain into 9 bilateral ROIs: caudate, cerebellum, frontal lobe, insula, occipital lobe, parietal lobe, putamen, temporal lobe, and thalamus. We then extracted the regional ΔTE metrics and averaged over the electrodes and voxels coinciding within the ROIs. To infer the net direction of the information transfer, we took the difference as $\Delta TE\left(x,y\right)=TE\left(x,y\right)-TE\left(y,x\right)$.

### Vasomotor Wave Propagation Speed.

To investigate vasomotor wave propagation within the human brain, we applied optical flow analysis to the MREG data, focusing specifically on infraslow oscillatory components associated with vasomotor activity. Optical flow analysis has been previously used for tracking motion within imaging data for different physiological pulsations including cardiac (60) and respiratory pulsations (61). By leveraging this method, we aimed to capture the spatiotemporal dynamics of vasomotor waves (31). We quantified the velocity and spatial extent of vasomotor wave propagation, thereby characterizing how these waves propagate across cortical and subcortical regions.

Pulse-triggering was guided by identifying regions demonstrating the highest correlations with vasomotor activity, following established methodologies. Specifically, the posterior cingulate cortex emerged as the region most strongly associated with vasomotor oscillations, leading to its selection as the primary reference region for triggering vasomotor wave analysis. This approach provided a precise framework for delineating the onset and propagation of vasomotor oscillations within the brain’s functional architecture.

### Statistical Analysis.

We had not made a priori statistical power calculation to predetermine the sample size for this study but used the material at hand. The alpha level was set to 0.05 for all statistical tests. We investigated whether infraslow power differed between epochs of wakefulness and the combined sleep states (NREM-1/2). A Wilcoxon rank-sum test (two-tailed) was employed, with the null hypothesis being that awake and sleep epochs had equal infraslow power (Fig. 2*B*). The false discovery rate (FDR) was controlled using FDR correction (62) to account for multiple comparisons. To compare voxelwise group differences in TE, we conducted a randomization test with 5,000 permutations, shuffling the arousal state labels for awake; NREM-1, awake; NREM-2, and NREM-1; NREM-2 (Fig. 3 *B* and *C*). Here, we used the threshold-free cluster enhancement method to address multiple comparisons. In comparison of whole-brain average TE and within ROI TE, we used a Wilcoxon rank-sum test (two-tailed), with the null hypothesis being that arousal states had equal median information transfer (Fig. 3 *B*–*E*). Here as well, we applied correction for the FDR. For comparison of BOLD power and speed maps between awake and sleep states, we used a randomization test along with threshold-free cluster enhancement (31).

## Supplementary Material

Appendix 01 (PDF)

## Data Availability

Analysis scripts, processed data files data have been deposited in GitHub (https://github.com/TommiVayrynen/Sleep-Alters-Neurovascular-and-Hydrodynamic-Coupling-in-the-Human-Brain). Some study data are available. The Finnish Act on the Secondary Use of Health and Social Data, along with the European union’s General Data Protection Regulation (GDPR), restricts data sharing when individuals could potentially be identified. Data sharing is possible upon request through collaborative research efforts, which are subject to agreements of further data sharing, analysis and publication. Previously published data were used for this work (Original dataset published in ref. 14).

## References

[1] S. Ogawa , Intrinsic signal changes accompanying sensory stimulation: Functional brain mapping with magnetic resonance imaging. Proc. Natl. Acad. Sci. U.S.A. **89**, 5951–5955 (1992). 1631079 10.1073/pnas.89.13.5951 PMC402116

[2] C. Kjaerby , Memory-enhancing properties of sleep depend on the oscillatory amplitude of norepinephrine. Nat. Neurosci. **25**, 1059–1070 (2022). 35798980 10.1038/s41593-022-01102-9 PMC9817483

[3] L. Xie , Sleep drives metabolite clearance from the adult brain. Science **342**, 373–377 (2013). 24136970 10.1126/science.1241224 PMC3880190

[4] A. Osorio-Forero, N. Cherrad, L. Banterle, L. M. J. Fernandez, A. Lüthi, When the Locus Coeruleus speaks up in sleep: Recent insights. Emerging perspectives. Int. J. Mol. Sci. **23**, 5028 (2022). 35563419 10.3390/ijms23095028 PMC9099715

[5] N. L. Hauglund , Norepinephrine-mediated slow vasomotion drives glymphatic clearance during sleep. Cell **188**, 606–622.e17 (2025), 10.1016/j.cell.2024.11.027. 39788123 PMC12340670

[6] V. Borchardt , Inverse correlation of fluctuations of cerebral blood and water concentrations in humans. Eur. Phys. J. Plus **136**, 497 (2021).

[7] N. E. Fultz , Coupled electrophysiological, hemodynamic, and cerebrospinal fluid oscillations in human sleep. Science **1979**, 628–631 (2019). 10.1126/science.aax5440 PMC7309589 31672896

[8] P. A. R. Bork , Astrocyte endfeet may theoretically act as valves to convert pressure oscillations to glymphatic flow. J. R. Soc. Interface **20**, 20230050 (2023). 37434503 10.1098/rsif.2023.0050 PMC10336390

[9] Y. Gan , Perivascular pumping of cerebrospinal fluid in the brain with a valve mechanism. J. R. Soc. Interface **20**, 20230288 (2023). 37727070 10.1098/rsif.2023.0288 PMC10509587

[10] L. Bojarskaite , Sleep cycle-dependent vascular dynamics in male mice and the predicted effects on perivascular cerebrospinal fluid flow and solute transport. Nat. Commun. **14**, 953 (2023). 36806170 10.1038/s41467-023-36643-5 PMC9941497

[11] C. Chang , Tracking brain arousal fluctuations with fMRI. Proc. Natl. Acad. Sci. U.S.A. **113**, 4518–4523 (2016). 27051064 10.1073/pnas.1520613113 PMC4843437

[12] M. Fukunaga , Large-amplitude, spatially correlated fluctuations in BOLD fMRI signals during extended rest and early sleep stages. Magn. Reson. Imaging **24**, 979–992 (2006). 16997067 10.1016/j.mri.2006.04.018

[13] H. Helakari , Human nrem sleep promotes brain-wide vasomotor and respiratory pulsations. J. Neurosci. **42**, 2503–2515 (2022). 35135852 10.1523/JNEUROSCI.0934-21.2022 PMC8944230

[14] H. Helakari , Effect of sleep deprivation and NREM sleep stage on physiological brain pulsations. Front. Neurosci. **17**, 1275184 (2023). 38105924 10.3389/fnins.2023.1275184 PMC10722275

[15] S. G. Horovitz , Low frequency BOLD fluctuations during resting wakefulness and light sleep: A simultaneous EEG-fMRI study. Human Brain Mapp. **29**, 671–682 (2008). 10.1002/hbm.20428 PMC6871022 17598166

[16] Z. Liu, M. Fukunaga, J. A. de Zwart, J. H. Duyn, Large-scale spontaneous fluctuations and correlations in brain electrical activity observed with magnetoencephalography. Neuroimage **51**, 102–111 (2010). 20123024 10.1016/j.neuroimage.2010.01.092 PMC2847019

[17] L. Marshall, M. Molle, S. Michaelsen, H. L. Fehm, J. Born, Slow potential shifts at sleep–wake transitions and shifts between NREM and REM sleep. Sleep **19**, 145–151 (1996). 8855037 10.1093/sleep/19.2.145

[18] T. Väyrynen , Infra-slow fluctuations in cortical potentials and respiration drive fast cortical EEG rhythms in sleeping and waking states. Clin. Neurophysiol. **156**, 207–219 (2023). 37972532 10.1016/j.clinph.2023.10.013

[19] S. Monto, S. Palva, J. Voipio, J. M. Palva, Very slow EEG fluctuations predict the dynamics of stimulus detection and oscillation amplitudes in humans. J. Neurosci. **28**, 8268–8272 (2008). 18701689 10.1523/JNEUROSCI.1910-08.2008 PMC6670577

[20] S. Vanhatalo , Infraslow oscillations modulate excitability and interictal epileptic activity in the human cortex during sleep. Proc. Natl. Acad. Sci. U.S.A. **101**, 5053–5057 (2004). 15044698 10.1073/pnas.0305375101 PMC387372

[21] A. Osorio-Forero , Noradrenergic circuit control of non-REM sleep substates. Curr. Biol. **31**, 5009–5023.e7 (2021). 34648731 10.1016/j.cub.2021.09.041

[22] J. W. Antony , Sleep spindle refractoriness segregates periods of memory reactivation. Curr. Biol. **28**, 1736–1743.e4 (2018). 29804809 10.1016/j.cub.2018.04.020 PMC5992601

[23] V. Kiviniemi , Real-time monitoring of human blood-brain barrier disruption. PLoS One **12**, e0174072 (2017). 28319185 10.1371/journal.pone.0174072 PMC5358768

[24] J. Voipio, P. Tallgren, E. Heinonen, S. Vanhatalo, K. Kaila, Millivolt-scale DC shifts in the human scalp EEG: Evidence for a nonneuronal generator. J. Neurophysiol. **89**, 2208–2214 (2003). 12612037 10.1152/jn.00915.2002

[25] J. M. Besson , Correlations of brain d-c shifts with changes in cerebral blood flow. Am. J. Physiol. **218**, 284–291 (1970). 4982913 10.1152/ajplegacy.1970.218.1.284

[26] L.-F. Jiang-Xie , Neuronal dynamics direct cerebrospinal fluid perfusion and brain clearance. Nature **627**, 157–164 (2024). 38418877 10.1038/s41586-024-07108-6 PMC12054998

[27] P. L. H. Chong, D. Garic, M. D. Shen, I. Lundgaard, A. J. Schwichtenberg, Sleep, cerebrospinal fluid, and the glymphatic system: A systematic review. Sleep Med. Rev. **61**, 101572 (2022). 34902819 10.1016/j.smrv.2021.101572 PMC8821419

[28] N. Huotari , Sampling rate effects on resting state fMRI metrics. Front. Neurosci. **13**, 279 (2019). 31001071 10.3389/fnins.2019.00279 PMC6454039

[29] T. Myllylä , Assessment of the dynamics of human glymphatic system by near-infrared spectroscopy. J. Biophotonics **11**, e201700123 (2018). 28802090 10.1002/jbio.201700123

[30] M. Lobier, F. Siebenhühner, S. Palva, J. M. Palva, Phase transfer entropy: A novel phase-based measure for directed connectivity in networks coupled by oscillatory interactions. Neuroimage **85**, 853–872 (2014). 24007803 10.1016/j.neuroimage.2013.08.056

[31] A. Elabasy , Sleep Alters the Velocity of Physiological Brain Pulsations in Humans. *Adv. Sci.*, 10.1002/advs.202503745 (2026). PMC13045454 41632045

[32] E. M. C. Hillman, Coupling mechanism and significance of the BOLD signal: A status report. Annu. Rev. Neurosci. **37**, 161–181 (2014). 25032494 10.1146/annurev-neuro-071013-014111 PMC4147398

[33] M. Järvelä , Increased very low frequency pulsations and decreased cardiorespiratory pulsations suggest altered brain clearance in narcolepsy. Commun. Med. **2**, 122 (2022). 36193214 10.1038/s43856-022-00187-4 PMC9525269

[34] B. Li , Anti-seizure effects of norepinephrine-induced free fatty acid release. Cell Metab. **37**, 223–238.e5 (2025). 39486416 10.1016/j.cmet.2024.10.011

[35] N. K. Logothetis , The effects of electrical microstimulation on cortical signal propagation. Nat. Neurosci. **13**, 1283–1291 (2010). 20818384 10.1038/nn.2631

[36] M. H. Murdock , Multisensory gamma stimulation promotes glymphatic clearance of amyloid. Nature **627**, 149–156 (2024). 38418876 10.1038/s41586-024-07132-6 PMC10917684

[37] E. Kim, J. Van Reet, S.-S. Yoo, Cerebrospinal fluid solute transport associated with sensorimotor brain activity in rodents. Sci. Rep. **13**, 17002 (2023). 37813871 10.1038/s41598-023-43920-2 PMC10562378

[38] S. Holstein-Rønsbo , Glymphatic influx and clearance are accelerated by neurovascular coupling. Nat. Neurosci. **26**, 1042–1053 (2023). 37264158 10.1038/s41593-023-01327-2 PMC10500159

[39] E. A. Nagelhus, T. M. Mathiisen, O. P. Ottersen, Aquaporin-4 in the central nervous system: Cellular and subcellular distribution and coexpression with Kir4.1. Neuroscience **129**, 905–913 (2004). 15561407 10.1016/j.neuroscience.2004.08.053

[40] T. A. Longden , Capillary K+-sensing initiates retrograde hyperpolarization to increase local cerebral blood flow. Nat. Neurosci. **20**, 717–726 (2017). 28319610 10.1038/nn.4533 PMC5404963

[41] C. A. Wotton, C. D. Cross, L. K. Bekar, Serotonin, norepinephrine, and acetylcholine differentially affect astrocytic potassium clearance to modulate somatosensory signaling in male mice. J. Neurosci. Res. **98**, 964–977 (2020). 32067254 10.1002/jnr.24597

[42] X. Liu , Subcortical evidence for a contribution of arousal to fMRI studies of brain activity. Nat. Commun. **9**, 395 (2018). 29374172 10.1038/s41467-017-02815-3 PMC5786066

[43] S. J. van Veluw , Vasomotion as a driving force for paravascular clearance in the awake mouse brain. Neuron **105**, 549–561.e5 (2020). 31810839 10.1016/j.neuron.2019.10.033 PMC7028316

[44] Y.-E.S. Ju , Slow wave sleep disruption increases cerebrospinal fluid amyloid-β levels. Brain **140**, 2104–2111 (2017). 28899014 10.1093/brain/awx148 PMC5790144

[45] V. Kiviniemi , Ultra-fast magnetic resonance encephalography of physiological brain activity–Glymphatic pulsation mechanisms?. J. Cereb. Blood Flow Metab. **36**, 1033–1045 (2016). 26690495 10.1177/0271678X15622047 PMC4908626

[46] A. Rayshubskiy , Direct, intraoperative observation of ~ 0.1 Hz hemodynamic oscillations in awake human cortex: Implications for fMRI. Neuroimage **87**, 323–331 (2014). 24185013 10.1016/j.neuroimage.2013.10.044 PMC3961585

[47] H. H. Wang , Physiological noise in MR images: An indicator of the tissue response to ischemia? J. Magn. Reson. Imaging **27**, 866–871 (2008). 18383248 10.1002/jmri.21007

[48] T. Bolt , A parsimonious description of global functional brain organization in three spatiotemporal patterns. Nat. Neurosci. **25**, 1093–1103 (2022). 35902649 10.1038/s41593-022-01118-1 PMC13716133

[49] V. Korhonen , Synchronous multiscale neuroimaging environment for critically sampled physiological analysis of brain function: Hepta-scan concept. Brain Connect. **4**, 677–689 (2014). 25131996 10.1089/brain.2014.0258 PMC4238249

[50] J. Assländer , Single shot whole brain imaging using spherical stack of spirals trajectories. Neuroimage **73**, 59–70 (2013). 23384526 10.1016/j.neuroimage.2013.01.065

[51] T. Hugger , Fast undersampled functional magnetic resonance imaging using nonlinear regularized parallel image reconstruction. PLoS One **6**, e28822 (2011). 22194921 10.1371/journal.pone.0028822 PMC3237553

[52] M. Jenkinson, C. F. Beckmann, T. E. J. Behrens, M. W. Woolrich, S. M. Smith, FSL. Neuroimage **62**, 782–790 (2012). 21979382 10.1016/j.neuroimage.2011.09.015

[53] R. W. Cox, AFNI: Software for analysis and visualization of functional magnetic resonance neuroimages. Comput. Biomed. Res. **29**, 162–173 (1996). 8812068 10.1006/cbmr.1996.0014

[54] P. J. Allen, O. Josephs, R. Turner, A method for removing imaging artifact from continuous EEG recorded during functional MRI. Neuroimage **12**, 230–239 (2000). 10913328 10.1006/nimg.2000.0599

[55] P. J. Allen, G. Polizzi, K. Krakow, D. R. Fish, L. Lemieux, Identification of EEG events in the MR scanner: The problem of pulse artifact and a method for its subtraction. Neuroimage **8**, 229–239 (1998). 9758737 10.1006/nimg.1998.0361

[56] R. Oostenveld, P. Fries, E. Maris, J.-M. Schoffelen, Fieldtrip: Open source software for advanced analysis of MEG, EEG, and invasive electrophysiological data. Comput. Intell. Neurosci. **2011**, 1–9 (2011). 21253357 10.1155/2011/156869 PMC3021840

[57] M. Cope, D. T. Delpy, System for long-term measurement of cerebral blood and tissue oxygenation on newborn infants by near infra-red transillumination. Med. Biol. Eng. Comput. **26**, 289–294 (1988). 2855531 10.1007/BF02447083

[58] M. X. Cohen, Analyzing Neural Time Series Data (The MIT Press, 2014).

[59] T. Schreiber, Measuring information transfer. Phys. Rev. Lett. **85**, 461–464 (2000). 10991308 10.1103/PhysRevLett.85.461

[60] Z. Rajna , Cardiovascular brain impulses in Alzheimer’s disease. Brain **144**, 2214–2226 (2021). 33787890 10.1093/brain/awab144 PMC8422353

[61] A. Elabasy , Respiratory brain impulse propagation in focal epilepsy. Sci. Rep. **13**, 5222 (2023). 36997658 10.1038/s41598-023-32271-7 PMC10063583

[62] Y. Benjamini, Y. Hochberg, Controlling the false discovery rate: A practical and powerful approach to multiple testing. J. R. Stat. Soc. Ser. B **57**, 289–300 (1995).

