# Supplementary material for: Sleep alters neurovascular and hydrodynamic coupling in the human brain
Source: Proc Natl Acad Sci U S A. 2026 Mar 18;123(12):e2510731123. doi: 10.1073/pnas.2510731123 (PMC13012097; doi:10.1073/pnas.2510731123)
Supplement: Supplementary file 1 — Appendix 01 (PDF) [file pnas.2510731123.sapp.pdf]

PNAS

Supporting Information for
Sleep Alters Neurovascular and Hydrodynamic Coupling in the Human Brain

Tommi Väyrynen*[1,2,3], Johanna Tuunanen[1,2,3], Heta Helakari[1,2,3], Ahmed Elabasy[1,2,3], Vesa Korhonen[1,2,3], Niko Huotari[1,2,3], Johanna Piispala[2,3,4], Mika Kallio[2,3,4], Maiken Nedergaard[5,6] Vesa Kiviniemi*[1,2,3,7]

Corresponding authors: Tommi Väyrynen, Vesa Kiviniemi
Email: tommi.vayrynen@oulu.fi, vesa.kiviniemi@oulu.fi

This PDF file includes:

Figures S1 to S4
Table S1

Figures

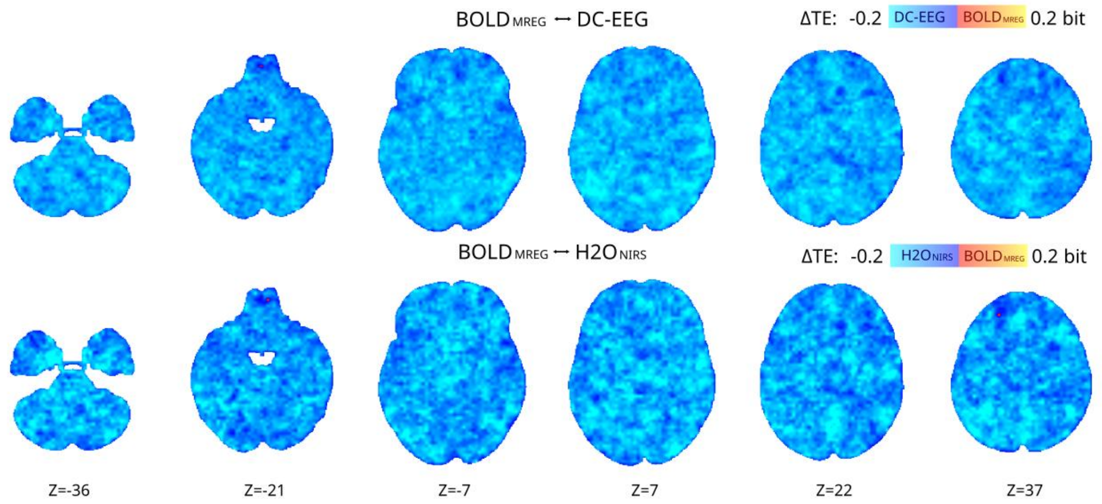

Figure S1. $BOLD_{MREG}$-EEG and $BOLD_{MREG}$-$H2O_{NIRS}$ phase transfer entropies (ΔTE) replotted from Figure 3, using a broader color scale range to visualize spatial differences. Negative values indicate that during the awake state, EEG and $H2O_{NIRS}$ signal changes predict $BOLD_{MREG}$ changes.

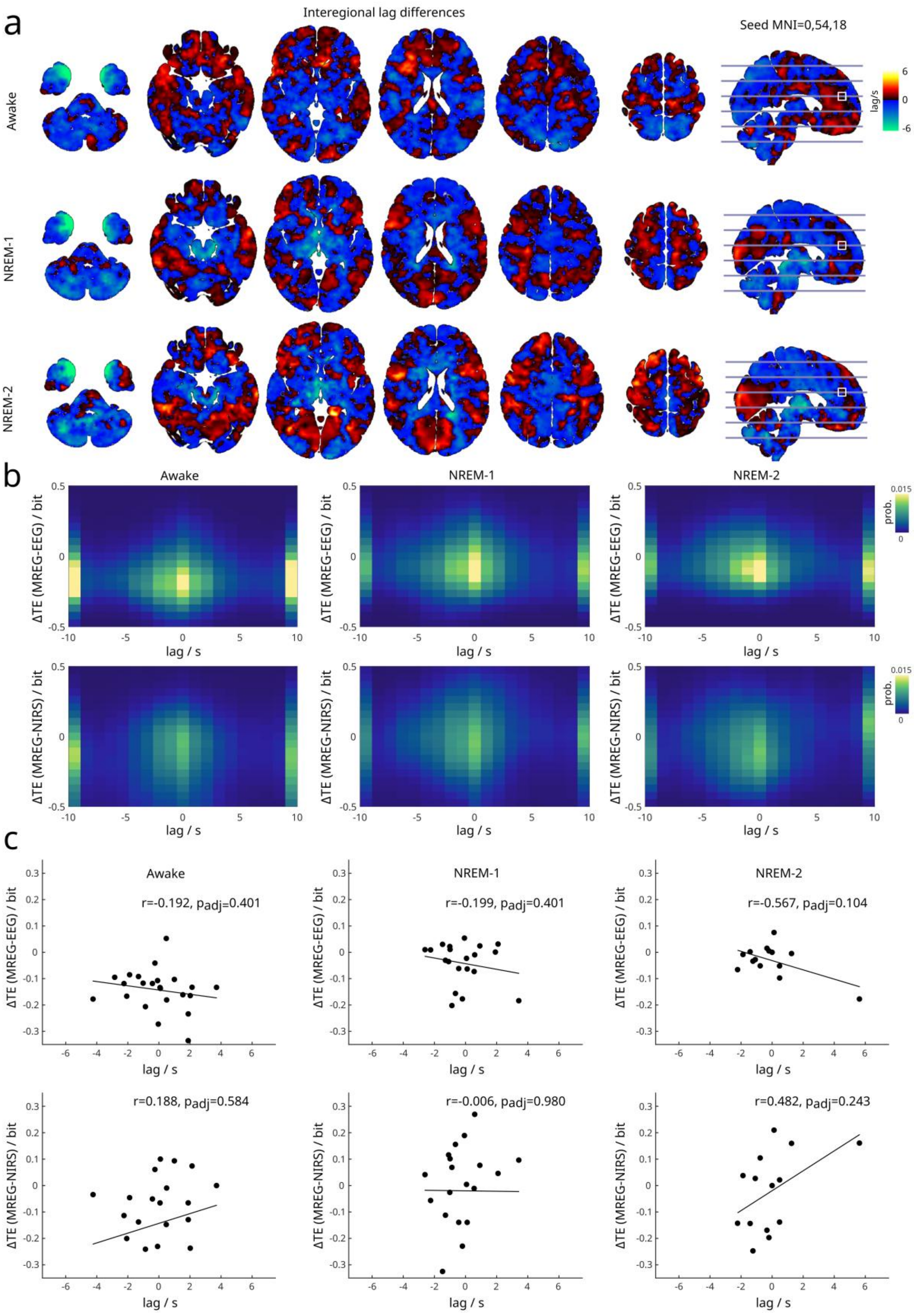

a
Interegional lag differences
Seed MNI=0,54,18
Awake
NREM-1
NREM-2
lag/s
6
0
-6
b
Awake
NREM-1
NREM-2
ΔTE (MREG-EEG) / bit
ΔTE (MREG-NIRS) / bit
lag / s
prob.
0.015
0
c
Awake
NREM-1
NREM-2
r=-0.192, padj=0.401
r=-0.199, padj=0.401
r=-0.567, padj=0.104
r=0.188, padj=0.584
r=-0.006, padj=0.980
r=0.482, padj=0.243
ΔTE (MREG-EEG) / bit
ΔTE (MREG-NIRS) / bit
lag / s

Figure S2. Seed-based inter-regional differences in $BOLD_{MREG}$ signal correlation levels are not correlated with TE values. (a) We used a seed-based approach to study the infra-slow correlation patterns within the MREG signal. Seed-voxel location on forehead was based on position of the NIRS optodes MNI(x,y,x)=[0,54,18], which was used to calculate cross-correlations with other voxels within the brain. Here, we allowed temporal lags of ±10 seconds in the search for maximum correlation. The delay (in seconds) corresponding to the maximum correlation was then extracted for each brain voxel. The process was repeated for all subjects and sleep states and averaged over the subjects. (b) We constructed bivariate histograms of inter-regional differences and the associated *ΔTE* for all voxels separately, to examine whether lag values depend on prediction values. The colour in each bin represents the probability of the corresponding values. (c) A scatter plot shows the mean whole brain lag values and *ΔTE* for each subject. We computed Pearson's linear correlation coefficient and the corresponding p-value, testing the null hypothesis that the two variables are uncorrelated. The p-values were adjusted for multiple comparisons using FDR correction. Ordinary least squares fit was applied to the data points for visualization purposes.

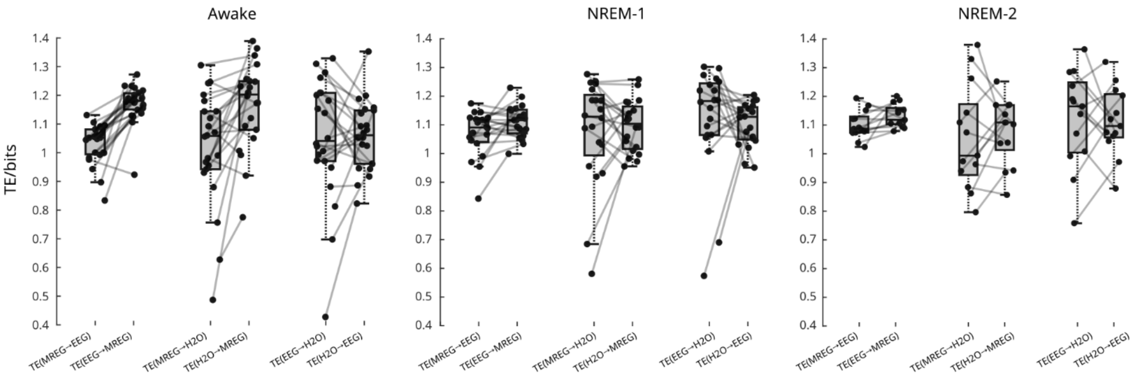

Figure S3. Separated $BOLD_{MREG}$-EEG and $BOLD_{MREG}$-$H2O_{NIRS}$ phase transfer entropies for both interaction directions: TE(x→y) and TE(y→x) in awake, NREM-1 and NREM-2 sleep states. Each dot represents the average TE for one subject taken over voxels and electrodes. Solid lines connect the paired values.

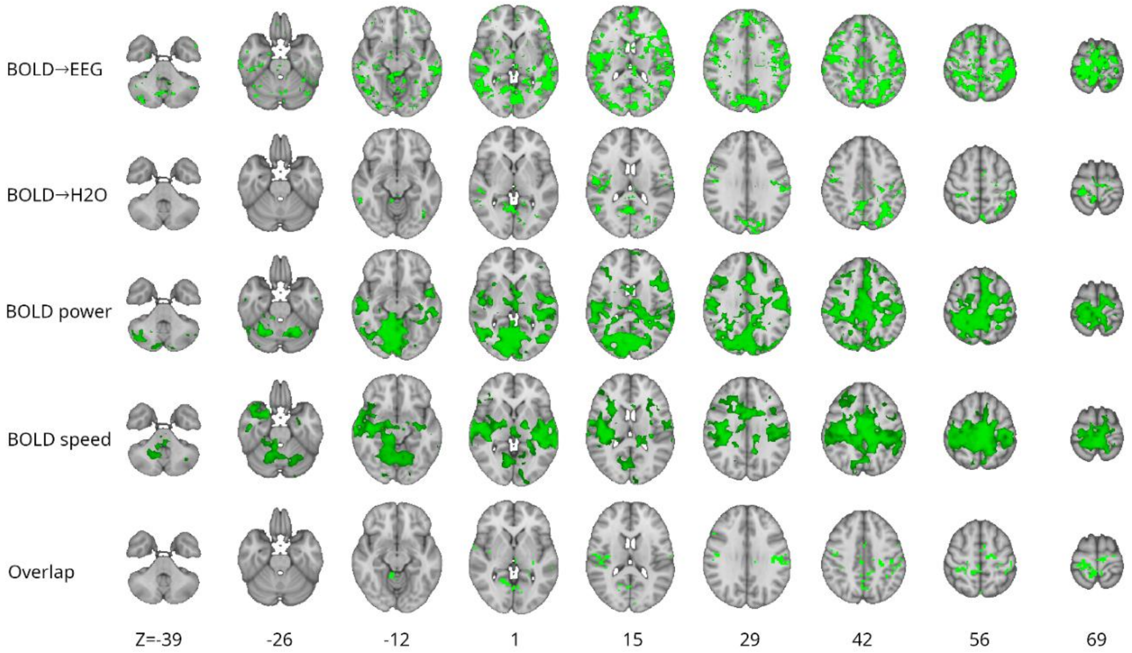

Figure S4. Statistically significant (sleep>awake, $p<0.05$) brain regions for sleep related changes in prediction patterns between: $BOLD_{MREG}$, EEG and $H2O_{NIRS}$ oscillations. Similarly, statistically significant BOLD power and BOLD vasomotor wave propagation speed increases are shown. The bottom row shows the overlap between these metrics. The z-coordinate is represented in MNI coordinate system.

Tables

Table S1. Region of interest analysis (ROI) of $BOLD_{MREG}$-EEG coupling (top) and $BOLD_{MREG}$-$H2O_{NIRS}$ (bottom). In the table median transfer entropy (*ΔTE*) values are shown for each ROI studied along with interquartile ranges. Statistical testing related *Z* and adjusted *p*-values (FDR-corrected) are shown as well.

MREG_BOLD - DC-EEG		Frontal lobe	Parietal lobe	Occipital lobe	Temporal lobe	Cerebellum	Caudate	Putamen	Insula	Thalamus
Med	A	-0.0864	-0.1236	-0.1331	-0.1035	-0.1026	-0.1244	-0.1220	-0.1183	-0.1241
ΔTE /bit	N1	0.0115	0.0023	-0.0188	-0.0159	-0.0298	-0.0175	-0.0278	-0.0291	-0.0329
	N2	0.0154	0.0157	-0.0179	-0.0180	-0.0059	-0.0179	-0.0061	-0.0070	-0.0068
IQR	A	0.0734	0.0713	0.0949	0.0648	0.0629	0.0791	0.0631	0.1014	0.0925
	N1	0.0864	0.1065	0.0855	0.0897	0.0940	0.1183	0.1243	0.1573	0.0957
	N2	0.0648	0.0714	0.0651	0.0872	0.0845	0.0331	0.0708	0.0906	0.0933
Z	A-N1	-3.4941	-3.7863	-3.4211	-3.4211	-2.8854	-3.0315	-2.8367	-2.6662	-3.0071
	A-N2	-3.5570	-3.5570	-3.8204	-3.4911	-3.3593	-3.9522	-3.7875	-3.6558	-3.9522
	N1-N2	0.3869	-0.2395	-0.6079	0.0553	-0.5711	-0.4974	-1.0500	-0.5711	-1.2711
$p_{adj.}$	A-N1	0.0013	0.0008	0.0014	0.0014	0.0066	0.0047	0.0072	0.0115	0.0047
	A-N2	0.0013	0.0013	0.0008	0.0013	0.0016	0.0008	0.0008	0.0012	0.0008
	N1-N2	0.7548	0.8419	0.6667	0.9559	0.6667	0.6963	0.3965	0.6667	0.2895
MREG_BOLD - H2O_NIRS		Frontal lobe	Parietal lobe	Occipital lobe	Temporal lobe	Cerebellum	Caudate	Putamen	Insula	Thalamus
Med	A	-0.1061	-0.1111	-0.0994	-0.1127	-0.0852	-0.1284	-0.1314	-0.1175	-0.1402
ΔTE /bit	N1	0.0263	0.0724	0.0056	0.0055	-0.0055	-0.0213	-0.0160	-0.0173	-0.0397
	N2	0.0271	0.0032	-0.0835	-0.0340	-0.0178	0.0006	0.0096	-0.0291	0.0427
IQR	A	0.2033	0.1872	0.2059	0.1766	0.1474	0.2931	0.2633	0.1892	0.1744
	N1	0.2083	0.2325	0.2258	0.2357	0.1574	0.2298	0.2259	0.2172	0.1830
	N2	0.2399	0.2675	0.2877	0.2202	0.2548	0.2923	0.3101	0.3113	0.2293
Z	A-N1	-2.5058	-2.7073	-2.3044	-2.0273	-1.7503	-1.6748	-1.8762	-1.9770	-1.8762
	A-N2	-1.5533	-1.8606	-1.5533	-1.2802	-1.6899	-1.3485	-1.3826	-1.6558	-1.8606
	N1-N2	0.3869	0.3132	0.1290	0.4974	0.0000	-0.0184	0.2395	-0.1290	-0.3500
p_{adj}	A-N1	0.1649	0.1649	0.1884	0.1884	0.2031	0.2031	0.1884	0.1884	0.1884
	A-N2	0.2166	0.1884	0.2166	0.3007	0.2031	0.2819	0.2814	0.2031	0.1884
	N1-N2	0.9255	0.9255	0.9692	0.8795	1.0000	1.0000	0.9517	0.9692	0.9255
